# Obesity and Diabetes in Pregnancy and Maternal and Child Health: The Gerar Cohort Profile, Brazil

**DOI:** 10.3390/healthcare14213783

**Published:** 2026-11-05

**Authors:** Mariana Pacheco de Oliveira, Samara Souza Stork, Anna Paula Piovezan, Anna Quialheiro, Gislaine Tezza Rezin, Betine Pinto Moehlecke Iser

**Affiliations:** 1 Graduate Program in Health Sciences, University of Southern Santa Catarina (UNISUL), Av. José Acácio Moreira, 787, Dehon, Tubarão 88704900, SC, Brazil; pachecomariana2405@gmail.com (M.P.d.O.); samarastork@gmail.com (S.S.S.); anna.piovezan@ulife.com.br (A.P.P.);; 2 Laboratory of Neurobiology of Inflammatory and Metabolic Processes, University of Southern Santa Catarina (UNISUL), Dehon, Tubarão 88704900, SC, Brazil; 3 Vale do Ave School of Health, Polytechnic Institute of Health of the North (CESPU), 4760-409 Vila Nova de Famalicão, Portugal; anna.quialheiro@ipsn.cespu.pt

**Keywords:** pregnancy, obesity, gestational diabetes, child development, maternal and child health

## Abstract

**Highlights:**

**What are the main findings?**

- The Gerar Cohort successfully implemented an integrated mother–child protocol, from first-trimester recruitment in primary care to child assessments at 12 months of age.
- One in four women entered pregnancy with pre-gestational obesity, and gestational diabetes mellitus was identified in 51 women, characterizing the cohort’s main exposure profile.

**What are the implications of the main findings?**

- The cohort’s design offers a replicable model for mother–child research across primary care and hospital settings, while its data and biospecimens—including placental tissue and oxidative stress biomarkers—provide a foundation for future studies on early-life health origins.
- Baseline characterization of obesity and GDM enables targeted subgroup analyses and may guide early preventive interventions during pregnancy.

**Abstract:**

Background: Pre-pregnancy obesity and GDM increase maternal–fetal oxidative stress, but their consequences for women’s health and child development are still unclear. Objectives: This paper describes the methodological design, recruitment strategy, data collection methods, and baseline characteristics of the Gerar Cohort, a prospective longitudinal study designed to investigate the relationship between pre-pregnancy obesity and gestational diabetes mellitus (GDM) with oxidative stress biomarkers and their effects on maternal and child health outcomes through 12 months postpartum. Methods: Women were recruited early in their prenatal care in a city in Southern Brazil. Sociodemographic and clinical data, as well as blood samples, were collected each trimester. At childbirth, umbilical cord blood and placental tissue were collected. Children were followed up until 12 months of age, with blood collection at 4 and 12 months. The study was approved by the Research Ethics Committee. Results: A total of 189 women were recruited in the first trimester; 136 were followed up until the third trimester; 23 pregnancy losses were recorded. Pre-gestational obesity was observed in 24.3% (95% CI 18.4–31.1), and 27% (95% CI 20.8–33.9) of women were diagnosed with gestational diabetes mellitus. Fifty-nine umbilical cord blood samples and 61 placental fragments were collected. In the children’s follow-up, 71 children were taken for ophthalmological examination at 1 month of age; 60 attended the pediatric consultation at 4 months; 56 children attended the neurological evaluation at 6 months and 50 at 12 months. Conclusions: The Gerar Cohort successfully implemented its recruitment and biospecimen collection procedures within public healthcare services.

## 1. Introduction

Pregnancy is a natural event that generally occurs without complications. However, due to pre-existing or acquired illnesses during the gestational period, a portion of women end up experiencing complications, both for themselves and for the fetus [1]. According to the Pan American Health Organization, 2026 [2], approximately 830 women die daily worldwide due to complications associated with pregnancy or childbirth. Among the main risk factors are maternal obesity and hyperglycemia, which increase perinatal morbidity and can cause negative impacts on the mother or the child in the medium and long term [3].

Obesity is considered one of the biggest public health problems in the world, and is classified as an epidemic that is increasingly advancing [4]. It is estimated that approximately 650 million adults worldwide live with obesity, and this number could exceed one billion individuals by 2030 if effective intervention measures are not implemented [5]. Obesity is characterized as a chronic inflammatory condition. During pregnancy, this inflammation can compromise fetal development and favor maternal–fetal complications, including epigenetic alterations, gestational complications, and metabolic disorders such as gestational diabetes mellitus (GDM), which is recognized as an important risk factor for adverse outcomes in maternal and neonatal health [6,7].

Diabetes is characterized by hyperglycemia resulting from alterations in insulin secretion and/or action, compromising the metabolism of carbohydrates, lipids, and proteins. It is estimated that approximately 537 million adults worldwide live with diabetes, and this number could reach 643 million by 2030 [8]. The increasing prevalence of this condition in women of childbearing age is noteworthy, given that the metabolic alterations related to glycemic dysfunction are among the most frequent complications during pregnancy. GDM can affect up to 18% of pregnancies, depending on the woman’s age and the diagnostic criteria used [9].

On the other hand, obesity, associated with this hyperglycemic environment, leads to an increase in oxidative stress and a reduction in antioxidant defenses, resulting in a number of complications, such as lipids, proteins, and deoxyribonucleic acid (DNA) damage [10,11]. Furthermore, the placenta is especially vulnerable; it can lead to complications during pregnancy, as well as a higher risk of DNA damage in newborns of mothers with obesity and/or GDM [11,12]. Given the global increase in obesity and diabetes in the adult population, it is essential to investigate how these conditions, when present before conception or diagnosed during pregnancy, affect women’s health during gestation, as well as child development.

The central hypothesis of the Gerar Cohort is that pre-pregnancy obesity and GDM increase maternal–fetal oxidative stress, with potential consequences for women’s health and child development; accordingly, the cohort’s primary objective is to evaluate the relationship between pre-pregnancy obesity and GDM with markers of oxidative stress. In addition to this primary focus, the cohort was designed to comprehensively characterize maternal and child health through complementary clinical, ophthalmological, neurodevelopmental, and lifestyle/psychological assessments, which, although secondary to the cohort’s central hypothesis, remain integral to a broader understanding of the health trajectories of mothers and children in this population. To this end, the Gerar Cohort prospectively followed pregnant women from the beginning of prenatal care, along with their live-born children through the first 12 months of life. The aim of this article is to describe the methodological design of the Gerar Cohort and to present initial results regarding sample composition.

## 2. Materials and Methods

This is a descriptive study of the methodology used in the prospective Gerar cohort, in which pregnant women were monitored from the beginning of prenatal care (first trimester of pregnancy) until the child’s first year of life. Data collection began in July 2023 and monitoring continued until August 2025 (Figure 1). This protocol was prepared following the STROBE guidelines [13] for observational studies, adapted for cohort profile reporting, as no dedicated reporting guideline exists for observational study protocols. The completed STROBE checklist is provided in the Supplementary Materials (Table S1).

### 2.1. Study Location

Data were collected from the Unidades Básicas de Saúde (UBS, Primary Health Care Units), university clinics, and a hospital in the municipality of Tubarão, located in the south of the state of Santa Catarina, Brazil. The municipality of Tubarão has 110,088 inhabitants, a Human Development Index (HDI) of 0.796, and a health infrastructure composed of 33 UBS and two hospitals [14]. The Nossa Senhora da Conceição Hospital (HNSC) is a regional reference center for high-risk pregnant women for 18 smaller municipalities in the region and provides free care through the Sistema Único de Saúde (SUS—Government Health System), registering between 250 and 300 births per month. Hence, this was the data source selected for this cohort study [15].

### 2.2. Study Population and Sample

The sample was composed of pregnant women in the first trimester of pregnancy, who received prenatal care at the municipality’s primary healthcare units between July and December 2023. Gestational age was estimated from the date of the last menstrual period and was confirmed by ultrasound.

Recruitment followed a census-based strategy: all eligible women initiating prenatal care at the municipality’s Basic Health Units were invited to participate during the study period. At the time of study planning, the Tubarão Municipal Health Foundation estimated that approximately 500 women were receiving prenatal care in these units. Because eligibility was restricted to women initiating prenatal care—approximately one-third of the caseload—the eligible population was estimated at approximately 166 women. Because the Foundation’s estimate referred to the prenatal caseload at a single point in time, the census-based strategy—which enrolled all women initiating prenatal care throughout the recruitment period—could yield a final sample exceeding this initial eligibility estimate.

Applying prevalence estimates from the current literature to this eligible pool, we expected to find approximately 39 women with pre-gestational obesity (based on Brazilian prevalence of obesity in women aged 18 to 34 years: 23.4%; 95% CI 16.6–31.8) [16] and 14 to 27 women with gestational diabetes mellitus (considering the regional estimates of GDM, which ranged from 8.7% to 16.7%, depending on the diagnostic criteria [17]. Co-occurrence of both conditions in the same woman was possible, although its proportion could not be anticipated. These expected numbers were compared with the minimum sample requirements for the main clinical outcomes (prematurity [18,19], and large-for-gestational-age infants [20,21,22], calculated using OpenEpi^®^ software (www.OpenEpi.com) with 80% power and a 95% confidence interval. Table S2 summarizes the parameters and results. With the conservative effect estimates from the current literature, the obesity-based clinical comparisons did not meet the minimum requirements and are considered exploratory, whereas GDM-based analyses are sensitive to the achieved sample size.

Because no published estimates of effect size or variability exist for continuous biomarkers of oxidative stress, sample size requirements were anchored on the clinical outcomes described above. Post hoc power for the main biomarker comparisons (DCF, SOD, and GSH) across the three BMI groups was estimated based on the achieved group sizes, using the approach for nonparametric tests described by Noether [23]. The medians and interquartile ranges observed in each group were converted to approximate standard deviations (SD ≈ IQR/1.35), from which an expected H statistic was derived and compared with the critical value of the chi-square distribution (α = 0.05, df = 2).

### 2.3. Data Collection

Data collection began in the first trimester of pregnancy, generally at the first prenatal visit at the primary healthcare unit, and continued throughout the gestational period, childbirth, and the follow-up of the child until they reached one year of age.

The questionnaires, composed of items related to anthropometric data, comorbidities, maternal gynecological and obstetric history, and infant information, were developed by the researchers and applied at different stages of the study (Figure 2). Data from each woman’s Electronic Health Record, maintained by the Municipal Health Foundation, were also verified, and hospital information was confirmed in the Philips Tasy^®^ system 5.04.1845177. Access to those data was obtained using a password assigned by the system administrators to the investigators, specifically for this purpose.

### 2.4. Prenatal Care and Pregnancy Monitoring

The recruitment of the participants took place in all the UBS in the municipality during the first-trimester prenatal visit. It was conducted by the research team, which was composed of college undergraduate and graduate students, with the assistance of service nursing professionals. All women who were in their first trimester of pregnancy were invited to participate in the study and received detailed information about the objectives of the research and all the necessary procedures. Those who agreed to participate registered their consent by signing the Informed Consent Form and/or the Informed Assent Form, in the case of pregnant minors.

Participants were classified according to pre-pregnancy body mass index (BMI) as eutrophic (18.5–24.9 kg/m^2^), overweight (25.0–29.9 kg/m^2^), or obese (≥30.0 kg/m^2^), according to the World Health Organization criteria [24]. Pre-pregnancy BMI was calculated using reported pre-pregnancy weight and measured height. When available, self-reported pre-pregnancy weight was cross-checked against the participant’s medical records. In cases of discrepancy, the weight recorded in the medical record was used for BMI classification. For participants aged younger than 18 years, body mass index-for-age z-scores were also calculated using the WHO [24] growth reference (5–19 years), with overweight defined as z-score > +1 and obesity as z-score > +2. The adult cut-offs were retained as the primary classification for the whole cohort, to preserve comparability across the sample.

GDM was identified retrospectively from three sources: fasting glucose values recorded in medical charts or prenatal cards; 75 g OGTT results when available; and GDM diagnosis recorded in the delivery record. Following the Brazilian Society of Diabetes (SBD) recommendation that pregnant women without a previous diagnosis of diabetes undergo fasting glucose testing at the first prenatal visit to detect overt diabetes or early GDM [7], women with first-trimester fasting glucose between 92 and 125 mg/dL were classified as having GDM. This approach was adopted because the 75 g OGTT is not always feasible in the Brazilian public health service [7,8]. When a 75 g OGTT was performed between 24 and 28 weeks of gestation, the International Association of Diabetes and Pregnancy Study Groups (IADPSGs) thresholds were applied: fasting plasma glucose ≥ 92 mg/dL, 1 h ≥ 180 mg/dL or 2 h ≥ 153 mg/dL, with a single abnormal value being sufficient for diagnosis. Because classification was retrospective, GDM status was assigned for the whole of pregnancy. Women who met diagnostic criteria at any point in their pregnancy—at the first prenatal visit or at the 24–28-week OGTT—were classified as having GDM. The GDM categories presented for the first trimester therefore reflect the final GDM status established during pregnancy and do not indicate that the diagnosis had already been made in the first trimester.

Pregnant women who did not understand Portuguese and/or presented alterations in comprehension or verbal expression that limited their ability to respond to the data-collection protocols were excluded from the study, as well as those who could not be recruited in the first trimester, or had gone through multiple pregnancies. Women who withdrew from participation at any point during the data-collection period, and those for whom delivery information was not available, were considered to be lost to follow-up. Pregnancy loss was defined as miscarriage or fetal death at any gestational age.

During the initial contact, all procedures and the duration of the study were presented. After signing the consent forms, a structured interview was conducted to collect clinical information and lifestyle habits (smoking), supplemented by data obtained from medical records and the pregnant women’s health booklet (Figure 2). At the same time, the first blood sample was collected. These steps were repeated in the second and third trimesters of pregnancy.

The blood samples were collected by nursing technicians and/or qualified nurses at three time points: first pregnancy trimester (up to 12 weeks), second trimester (18–24 weeks), and third trimester (32–35 weeks). Maternal samples were obtained by venipuncture in the forearm using a vacuum-collection system with a tube containing clot activator (8 mL capacity); a total of 4 mL of blood were collected at each stage. Subsequently, the samples were forwarded to the Neurobiology of Inflammatory and Metabolic Processes Laboratory (NEUROIMET), located at the university, for processing (maximum of 2 h after collection) and storage in an ultrafreezer at −80 °C.

During the follow-up period, the investigators maintained continuous contact with the participants through messaging, home visits, and communication with nursing teams at the UBS; this contact allowed the investigators to monitor possible gestational complications and identify potential losses to follow-up, such as miscarriage or other relevant clinical situations.

Pregnant women monitored at the UBSs were divided into six groups: women with pre-pregnancy obesity, women with pre-pregnancy overweight, women who developed gestational diabetes mellitus (GDM), women with overweight and associated GDM, women with obesity and associated GDM, and women without such comorbidities. Overweight and obesity were defined by the pre-pregnancy Body Mass Index (BMI), calculated based on the weight and height reported by the women at the first visit, referring to the period prior to pregnancy, and confirmed in their medical records. GDM was classified based on the medical registries of diagnosis or glycemic levels.

From the second trimester onwards, participants were invited to complete the FANTASTIC lifestyle questionnaire during prenatal appointments or through invitations sent via messaging app. The instrument, validated in Brazil, consists of 25 items grouped into nine domains: F = family and friends; A = activity; N = nutrition; T = tobacco and toxics; A = alcohol; s = Sleep, seatbelts, stress, and safe sex; T = type of behavior; I = insight; and C = career (job satisfaction). The score is classified, according to the number of points obtained, as excellent (85–100), very good (70–84), good (55–69), fair (35–54) and poor (0–34) [25,26].

The Edinburgh Postnatal Depression Scale (EPDS) was completed by the participating women in the third trimester of pregnancy and postpartum. The EPDS is a self-administered questionnaire indicated for screening for potential symptoms of depression. This instrument has 10 items, each with 4 possible answers, with the score associated with the severity of the symptom [27,28]. The sum of the points indicates a low risk of depression or a picture suggestive of postpartum depression, using a cut-off of ≥10 points to identify probable depression, in the version validated for the Brazilian population by Santos et al. [28].

### 2.5. Childbirth

To facilitate the identification of the study participants at the time of delivery, a sticker with the project logo was placed on the cover of the pregnant woman’s booklet. Thus, the research team was informed of the pregnant women’s admission to the HNSC via messaging app, either by the participant herself or by the obstetrics center’s nursing staff, who could then proceed with the collection and processing of the biological samples.

At birth, umbilical cord blood (2 mL) was collected by the obstetric center team shortly after delivery, through venipuncture and then transferred to a tube with a previously identified clot activator. The placentas were collected at the end of their delivery; they were sectioned with the aid of a scalpel blade, respecting a minimum margin of 2 cm from the insertion of the umbilical cord and the edge, covering regions representing the maternal–fetal interface (decidua basalis, chorionic villi and amniotic plate). The placental fragment had the amniotic plate dissected from the trophoblast and the resulting portions—membrane, fetal side and maternal side—were transferred to 2 mL microtubes, as well as 2 cm of the umbilical cord. Ribonucleic acid (RNA)later was added to the microtubes containing the samples in a minimum volume of 10× the weight of the tissue. The collection of umbilical cord blood and tissue from the umbilical cord and the placenta was carried out by the nursing team that was attending the birth, who had prior agreement and training; subsequently, the material was forwarded to the NEUROIMET laboratory, located at the university, for processing of the umbilical cord blood and storage of both samples in the ultrafreezer −80 °C (maximum of 2 h after collection).

In the laboratory, placental and umbilical cord samples stored in RNAlater remained in the ultrafreezer until the end of the deliveries and were then forwarded to the Center for Biochemical Pharmacology at the William Harvey Research Institute, London, UK, for analysis.

### 2.6. Postpartum Monitoring and Child Development

The live-born babies of the participating women who gave birth at the HNSC were followed up with in-person assessments conducted at the university outpatient clinic at 1, 4, 6, and 12 months of age; the visits were scheduled through contact with the mothers.

In the first month, an appointment with an ophthalmologist was arranged to assess lens transparency, retinal vessel formation, and the possibility of retinopathy, generally linked to prematurity. The mothers received guidance and were given information regarding any queries about childcare. In addition, the participants again filled out the EPDS [27,28], a questionnaire administered under the supervision of a psychology professional, to screen for symptoms of postpartum depression and compare it with their emotional state in the third trimester of pregnancy.

In the fourth month, the babies were seen by medical interns in a pediatric visit under the supervision of a pediatric gastroenterologist. Weight gain (weight and height), breastfeeding and introduction of solid foods, and gastrointestinal health were assessed.

In children, at six months of age, a neuropsychomotor development assessment was conducted using developmental milestones recommended by the Brazilian Society of Child Neurology, based on the Act Early program of the Centers for Disease Control and Prevention. This instrument consists of a structured checklist of expected milestones for the age, allowing identification of whether the child presents the expected skills for their age group and possible signs of developmental delay [29].

The assessment was conducted by medical students who had been previously trained and was performed under the supervision of a physician specializing in pediatric neurology. During this phase, the child’s eating habits and gastrointestinal health were also evaluated. At that time, the mothers were invited again to fill out the FANTASTIC questionnaire, in order to compare their prenatal habits with their postpartum-period habits [25,26].

The follow-up continued until the child was 12 months old, at which time a neurodevelopmental assessment was conducted using the Bayley Scales of Infant Development (Bayley-III). This instrument is validated for assessing the development of children aged 1 to 42 months, with adaptations for chronological age or corrected age in cases of preterm birth. The instrument contains 136 items distributed across specific subscales; the scale analyzes sensorimotor skills, memory, receptive and expressive communication, and fine and gross motor skills. Based on the scores obtained during the assessment, it is possible to identify developmental delays and direct interventions, when necessary, according to standardized guidelines and normative tables [30,31].

Assessments were applied by undergraduate research assistants under the direct supervision of a pediatric neurologist certified in the administration of the Bayley-III, along with two additional physicians trained by the certified assessor. All supervising and assisting personnel underwent an 8 h training session prior to each application, conducted and directly supervised by the certified pediatric neurologist, to ensure standardized administration procedures.

It is worth highlighting that after the visits at 4 months and 12 months of age, the child’s blood was collected from a forearm vein using a 5 mL syringe; 2 mL blood was collected. Both collections were performed by a qualified professional with experience in pediatric blood collection. After collection, the blood was immediately transferred to a tube with a previously identified clot activator.

At each stage of the research, the collected material was transported in an ice-containing styrofoam case to NEUROIMET for processing within a maximum of 2 h after collection. The blood samples were initially centrifuged (3500 rpm for 10 min) and the serum was separated into 2 mL microtubes. The material was stored in an ultrafreezer at −80 °C until the corresponding analyses were performed.

### 2.7. Biochemical Analyses

Maternal blood samples were collected during the first, second and third trimesters of pregnancy, between July 2023 and July 2024, and were shipped to the analysis laboratory in batches after completion of the maternal collection phase; consequently, storage duration at −80 °C prior to analysis ranged from approximately 1 to 13 months, depending on each participant’s collection date. Delivery-related samples (umbilical cord blood and placental tissue) were collected between January and August 2024 and followed the same batch-shipment procedure, with storage duration ranging from approximately 1 to 8 months. Child blood samples, collected during the 4-month (May to December 2024) and 12-month follow-up visits (January to August 2025), were stored for approximately 1 to 8 months before analysis.

Blood samples collected from pregnant women in the first, second, and third trimesters of pregnancy, as well as umbilical cord blood and blood from children at 4 and 12 months of age, were analyzed for concentrations of oxidative stress biomarkers, including dichlorofluorescein (DCF) and reduced glutathione (GSH); superoxide dismutase (SOD) activity was measured at the University of Southern Santa Catarina (UNESC). Biochemical analyses were performed in a subsample of participants rather than in the entire cohort. A target of 20 participants per group was established a priori for planning purposes, considering the six groups defined by BMI category (eutrophic, overweight, and obesity) and the presence or absence of gestational diabetes mellitus (GDM). In groups with fewer than 20 eligible specimens, all available samples were processed. In groups with a minimal excess over the target (*n* = 21), all available samples were also processed. In the group with the largest number of eligible specimens, the selection prioritized those with the most complete follow-up, based on the number of collections performed up to the fourth month of the child’s life.

The analysis of reactive oxygen species (ROS) production was determined by the intracellular formation of DCF. Initially, the samples were homogenized, then 2.7-dichlorodihydrofluorescein diacetate was added, and the samples were subsequently incubated for 30 min at 37 °C. Fluorescence was measured in a SpectraMax^®^ iD3 Multi-Mode Microplate Reader (Molecular Devices, San Jose, CA, USA) using a 488 nm excitation wavelength and a 525 nm emission wavelength, with a calibration curve generated using standard DCF (0–10 µM). The results were expressed as nmol DCF/mg protein [32].

The analysis of antioxidant enzyme activity of SOD was determined by the inhibition of adrenaline auto-oxidation, measured spectrophotimetrically. The technique is based on the inhibition of the reaction between the O_2_•^−^ radical and adrenaline, a compound that auto-oxidizes with pH variation. The oxidation of adrenaline leads to the formation of adrenochrome, and the activity of SOD was determined by using the SpectraMax^®^ iD3 Multi-Mode Microplate Reader (Molecular Devices, San Jose, CA, USA) to measure the rate of adrenochrome formation at 480 nm, in a reaction medium containing glycine–NaOH (50 mM at pH 10.2) and 60 mM adrenaline. The results were expressed in U/mg of protein [33].

The analysis of GSH levels was measured after protein precipitation with 1 mL of 10% trichloroacetic acid. Phosphate buffer (800 mM, pH 7.4) and 500 µM of 5.5′-dithiobis-(2-nitrobenzoic acid) (DTNB) were then added. The color development resulting from the reaction between DTNB and thiols peaked at 5 min and remained stable for more than 30 min. Absorbance was measured at 412 nm after 10 min in a SpectraMax^®^ iD3 Multi-Mode Microplate Reader (Molecular Devices, San Jose, CA, USA).. A standard GSH curve was used to calculate its levels in the samples. The results were expressed in µmol/mg of protein [34].

All blood analyses were normalized for protein content. Proteins were determined by the method of Lowry et al. (1951), using bovine serum albumin as a standard [35].

Ribonucleic acid (RNA) from dissected placental tissue was extracted using the Rneasy Mini Kit, following the manufacturer’s instructions. RNA quality and concentration were verified by absorbance in nanodrops before reverse transcription, performed with the iScript™ cDNA synthesis kit, according to the manufacturer’s instructions. qPCR was performed with 10 ng of cDNA, 1 µm primers for tnf-α, il-1β, cd45, and il-10, and 1x SYBR Green qPCR Master Mix, in a Cfx connect LightCycler thermal cycler. Gene expression was calculated using the method of Vandesompele [36], with normalization using the reference genes top1 and cyc1. The cycling conditions were as follows: 95 °C for 30 s, followed by 40 cycles at 95 °C for 5 s and at 60 °C for 30 s, with analysis of the dissociation curve at the end. All experiments were performed in duplicate [36].

### 2.8. Statistical Methods

The data collected were entered into the Jamovi statistical software version 2.7.9 (Jamovi Project, Sydney, Australia). Quantitative variables were described using measures of central tendency and dispersion (mean ± standard deviation—SD; median and interquartile range—IQR), according to normality assessed by the Shapiro–Wilk test. Categorical variables were reported as frequencies and percentages, and prevalence estimates are presented with 95% confidence intervals (95% CIs), calculated using he Clopper–Pearson exact method. Exploratory comparisons were performed to characterize the sample according to groups of interest, considering pre-pregnancy obesity and GDM as the main exposure; Chi-square or Fisher’s exact test were used for categorical variables. Continuous variables with non-normal distributions were compared using the Mann–Whitney U test (two groups). Baseline characteristics were also compared between women retained to follow-up and those lost to follow-up, and between women who experienced pregnancy loss and those with ongoing pregnancies, using the same tests. Biochemical variables were analyzed using the two-way analysis of variance (ANOVA) followed by Tukey’s post hoc test, as an exploratory approach. The significance level set for the statistical test was 5% (*p* < 0.05).

### 2.9. Planned Analyses

For each study derived from this cohort, specific multivariate analysis models and corresponding measures of association will be determined based on the outcomes of interest. Planned analyses will address the effects of pre-pregnancy BMI and GDM on biomarker trajectories throughout pregnancy, birth outcomes, and child development, incorporating repeated measurements through linear mixed-effects models (with generalized linear mixed models for non-Gaussian outcomes), which appropriately handle within-subject correlation across gestational time points. Mediation analysis will be employed to evaluate whether biomarker changes mediate the association between maternal exposures and child outcomes. Maternal age and pre-pregnancy BMI will always be considered potential confounders, with additional covariates selected according to each outcome. Missing data will be handled according to the assumed mechanism: mixed-effects models provide valid inference under the missing-at-random assumption, and multiple imputation will be considered where appropriate.

## 3. Preliminary Results

The research team sought to recruit all pregnant women who were starting prenatal care at the 33 UBSs in the municipality between July and December 2023. A total of 191 pregnant women were interviewed, and after two exclusions, 189 women continued in the study, exceeding the initial eligibility estimate of approximately 165, as the census captured all women initiating prenatal care during the recruitment period. Figure 3 below explains the behavior of the sample in each phase of the data collection, from the mother’s initial recruitment to the monitoring of the child at 12 months of life.

The median age of participants was 27 years (IQR 10), ranging from 14 to 46 years; 77% were aged 20–34 years, 34% were primigravidas (in their first pregnancy) and 39% were nulliparous (with no previous deliveries), as shown in Table 1.

### 3.1. Monitoring During Prenatal Care According to the Interest Groups

Out of the total number of women included in the study, 46 were obese (24.3% 95% CI 18.4–31.1), and 54 (28.6% 95% CI 22.2–35.6) were overweight. GDM was identified in 51 women (27.0%; 95% CI 20.8–33.9). Of these, 25 (49.0%) were identified in the first trimester based on fasting glucose values, and six (11.8%) had their diagnosis ascertained from the delivery record without an available glucose value. Laboratory confirmation was therefore available for 45 cases (88.2%). The sample composition according to BMI classification and GDM is presented in Table 2. As pregnancy progressed, some losses occurred: 23 pregnancy losses (miscarriage or fetal death) were registered (12.2% 95% CI 7.8–17.7). One fetal death occurred at 35 weeks of gestation, and was attributed to hypertensive complications. Women who experienced pregnancy loss were older than those with ongoing pregnancies (median age 33 years [P25–P75: 25.0–36.5] vs. 27 years [P25–P75: 21.3–31.0]; *p* = 0.009, Mann–Whitney U test). Pregnancy loss occurred in 12.4% of women with adequate nutritional status, 9.3% of those with overweight, and 15.2% of those with obesity (Table 2), with no statistically significant difference among groups (*p* = 0.657 Fisher’s exact). The third trimester was completed by 136 women (71.9%), all of whom had at least one blood sample collected during this period (Figure 3).

Of the 136 women who remained in follow-up through the third trimester, one fetal death at 35 weeks of gestation and 20 losses of contact were registered; delivery information was available for 115 (85.2%). The biochemical analytical sample comprised 11 to 21 participants per group across the six groups defined by BMI category and GDM status: eutrophic without GDM (*n* = 20), eutrophic with GDM (*n* = 20), overweight without GDM (*n* = 21), overweight with GDM (*n* = 16), obesity without GDM (*n* = 21), and obesity with GDM (*n* = 11), totaling 109 participants. Umbilical cord blood and placental tissue were collected in at least 59 deliveries, corresponding to 51.0% of those with available delivery information. The collections are described by interest group, as shown in Table 3.

### 3.2. Child Monitoring, According to the Interest Groups

Table 4 presents the total number of children who attended each time phase (first, fourth, sixth, and 12th months of life) and the respective blood samples collected from the children at the fourth and 12th month, according to the interest groups. Because the number of eligible participants differed between groups, the analytical sample in the biochemical analyses comprised a different number of participants in each group. Post hoc power analysis based on the achieved sample sizes indicated power exceeding 99% for DCF and SOD and approximately 85% for GSH across the three BMI groups, indicating adequate capacity to detect the differences in interest in the main biochemical outcomes. Power estimates for the additional comparisons (by GDM status and across the six groups) will be reported in the respective specific analyses.

Baseline characteristics measured at enrollment—maternal age, pre-pregnancy BMI and its classification, and marital status—were compared between women retained to the third-trimester assessment (*n* = 135) and those lost to follow-up (*n* = 31), and no statistically significant differences were identified in these main characteristics (Table 5). Mothers of children not followed up to 12 months were younger than those of children retained in follow-up (median 26 years, IQR 21–30 vs. 29.5 years, IQR 25–33,8; *p* = 0.005, Mann–Whitney test) (Table 6).

## 4. Discussion

This study initially included 189 pregnant women in their first trimester of pregnancy who attended the 33 UBSs in the municipality; they were monitored until delivery and their children were followed up for the first year of life. Data collection was maintained across all predefined time points, yielding an integrated dataset that combines clinical and sociodemographic information with biological samples from both mothers and their children. The same dyads were assessed repeatedly, which allows maternal exposures, pregnancy outcomes and infant outcomes to be analyzed within a single linked framework.

To support engagement and retention, complementary strategies were implemented. A voluntary peer-support group was created on a smartphone messaging application and linked to a university outreach project on self-care, quality of life and maternal mental health, through which participants exchanged experiences and received educational content on nutrition, breastfeeding and prenatal care. This channel also served as a direct route for health guidance and appeared to strengthen participants’ connection with the study. At delivery, participant identification was facilitated by stickers affixed to the prenatal care cards, with the collaboration of hospital nursing staff, and many women notified the study team directly upon admission.

Losses occurred at all study stages and were substantial. Of the 189 women included, 23 (12.2%) experienced pregnancy loss, 31 (16.4%) were lost to follow-up during pregnancy, and 20 (14.8% of those who reached the third trimester) had no delivery information available—mostly owing to loss of contact, so the reasons for these losses could not be evaluated. Umbilical cord blood and placental tissue were collected in 59 (51%) and 61 (53%) of the 115 deliveries with available information. Although at-delivery biospecimen collection was planned for all participants, it could not always be performed because of occasional staff shortages and the workload of the obstetric center at certain times, as well as the mothers’ own clinical conditions at delivery. During the postnatal follow-up, participation declined further—follow-up at 12 months was achieved for 50 children, corresponding to 26.5% of the original cohort and 43.5% of those with delivery information. Return to work was frequently mentioned by participants as a reason for missing appointments, even though visits were offered most frequently at the end of the day and on Saturdays; however, the reasons for non-attendance were not systematically documented, and several of these losses corresponded to loss of contact rather than explicit withdrawal. These operational experiences may inform the design of future mother–child cohort studies in similar settings. The comparison between participants retained and those lost (Table 5 and Table 6) showed no difference other than maternal age, which was higher among mothers who attended the 12-month assessment. Residual selection bias, however, cannot be excluded and is stated as a limitation. It is important to note that the 23 pregnancy losses constitute a study outcome rather than attrition, and that the reasons for loss differed across study phases. Nonetheless, the cumulative reduction in sample size limits the precision of the infant outcome analyses and should be considered when interpreting the findings.

The observed proportion of pregnancy losses (12.2%; 95% CI 7.8–17.7) was compatible with international estimates indicating that 10–25% of pregnancies end in miscarriage, generally in the first trimester [37]. However, reliable official data miscarriage in the Brazilian population are lacking [38], as not all pregnancy losses are documented, which limits formal comparison. The exact timing and causes of pregnancy losses could not be ascertained. Women who experienced pregnancy loss were older than those with ongoing pregnancies, a finding consistent with the established association between advanced maternal age and pregnancy loss [39], which supports the internal validity of the cohort data. In contrast, younger women were less likely to remain in follow-up, indicating differential attrition. Because these two processes removed women from opposite ends of the age distribution, their effects on the age composition of the final sample may partially offset each other. Loss to follow-up was progressive: of the 189 women enrolled, 135 (71.4%) completed the third trimester, 115 (60.8%) had a delivery with eligible sample collection, and 50 children (26.5%) were assessed at 12 months. Part of this reduction is inherent to outcome ascertainment rather than to participant withdrawal, but differential loss by maternal age was observed and may introduce selection bias in analyses of maternal and child outcomes, that should be considered when interpreting the findings. In contrast, losses to follow-up were not associated with baseline characteristics, suggesting limited attrition bias.

The data found so far allow us to estimate the prevalence of health conditions in the sample: obesity values were similar to those indicated in the literature for Brazilian women but were higher than predicted for GDM [9,16]. It is possible that women who were known to be at risk of developing chronic diseases had greater adherence to the prenatal assessment and to the study, increasing their prevalence. In any case, the prevalence of these conditions should serve as a warning sign to the health system, considering the known risks that these conditions, especially when present during pregnancy, can bring to the health of women and to their offspring [40].

This prevalence of obesity and GDM reinforces the relevance of the oxidative stress pathway investigated by this cohort, given the established role of both conditions in amplifying maternal–fetal reactive oxygen species production and impairing placental function [41,42]. The Gerar Cohort was specifically designed to capture this oxidative stress allowing future analyses to directly test these biological pathways. As maternal age was associated with pregnancy loss in this cohort, it will be treated as a potential confounder in future outcome analyses.

Few cohorts have investigated the joint effects of pre-pregnancy obesity and GDM on maternal and offspring health. The Xi’an Mother–Child Cohort (XAMC) in China, for instance, examined these associations retrospectively [43]. However, these cohorts have primarily focused on anthropometric and glycemic outcomes, with oxidative stress discussed as a potential underlying mechanism rather than measured directly. The Gerar Cohort addresses this gap by collecting oxidative stress biomarkers at multiple time points across pregnancy (during the first, second, and third trimesters), at delivery (placental tissue and umbilical cord blood), and during early childhood (4 and 12 months), enabling group comparisons (e.g., obesity/non-obesity, GDM/non-GDM) at each of these stages.

### Strengths and Limitations

This study has several strengths. The first was the prospective design, initiated in the first trimester of pregnancy and extending through the first year of the child’s life, facilitated data collection at multiple key time points across pregnancy, delivery, and early childhood. The second was the cohort’s biological sampling strategy, encompassing maternal blood across three trimesters, umbilical cord blood, placental tissue, and child blood at 4 and 12 months. Thirdly, recruitment was carried out at all primary healthcare units in the municipality, reflecting the real-world conditions of Brazil’s public health system and enhancing the practical relevance of the operational findings for similar settings. Finally, the support strategies developed during follow-up, including messaging-app support groups and a maternal mental health extension project, represent an additional contribution of this study, offering both a replicable retention strategy and a direct channel of care for participants.

By measuring placental biomarkers and oxidative stress markers in maternal blood, in cord blood, and in placental tissue obtained at delivery, our cohort characterizes oxidative stress in both compartments within the same mother–infant pair, alongside pre-pregnancy anthropometry and detailed maternal metabolic characterization. This design will allow us to examine whether placental oxidative stress mediates the association between pre-pregnancy obesity, GDM, and adverse maternal and child outcomes, rather than merely documenting systemic redox imbalance.

This study has some limitations that should be considered. The sample size was determined by the accessible population through a census-based recruitment strategy, rather than by a formal recruitment target; consequently, the statistical power of the planned analyses was verified a priori against literature-based expectations rather than guaranteed by design. With conservative effect estimates derived from the current literature (converted from adjusted odds ratios to risk ratios), the expected number of women with pre-gestational obesity (approximately 39) did not meet the minimum requirements for the obesity-based clinical comparisons (preterm birth and large-for-gestational-age infants); these analyses should therefore be interpreted as exploratory and hypothesis-generating. Analyses involving gestational diabetes mellitus met the minimum requirement marginally across the expected prevalence range, with a comfortable margin only at the upper range, and are therefore sensitive to the achieved sample size. In addition, the co-occurrence of obesity and GDM in the same women, although possible, could not be anticipated in the planning stage and may reduce the effective numbers available for some comparisons. Analyses involving the 50 children assessed at 12 months of age have larger minimum detectable effect sizes. The reasons for losses to follow-up were not systematically recorded, so that active withdrawal could not be distinguished from loss of contact. The factors described here reflect the study team’s operational experience rather than a formal analysis of attrition determinants and should be interpreted as potential mechanisms rather than established causes.

The representativeness of the cohort in relation to the broader pregnant population of Tubarão cannot be fully ascertained, as no municipal registry provides the exact number of women initiating prenatal care during the study period. Publicly available data from the national Live Birth Information System (SINASC) offer only partial context: approximately 100 births per month were recorded in the period comprising the births of the cohort; however, the figures do not distinguish between the two hospitals in the city or between public and private sector care, and, although live births were selected by maternal residence, Tubarão is a referral center for 18 smaller municipalities in the region, which may inflate the total number of births recorded. Notably, 92% of live births recorded in Tubarão in the study period were preceded by prenatal care initiated in the first trimester, suggesting that the proportion of women beginning prenatal care later in pregnancy is small. Nevertheless, women who begin prenatal care later, or who decline to participate in research, may differ systematically from those included in this cohort, which could affect the prevalence estimates of pre-pregnancy obesity and GDM observed in this sample. Within these limitations, according to SINASC data, 71.5% of pregnant women are aged 20 to 34 years, broadly similar to the age distribution observed in the cohort (77%). Information on the prevalence of obesity and GDM in the local pregnant population is not available in the national system, which precluded a formal comparison for these conditions—although it also positions the present cohort as the first local source of estimates for their frequency in the region. As a single-municipality cohort recruited in primary care settings, the generalizability of the findings to other contexts should be considered with caution.

Body mass index was classified using the World Health Organization adult cut-offs for all participants. Nine participants (4.8%) were aged 14–17 years at enrolment, for whom adult cut-offs may misclassify nutritional status. When these participants were classified according to the WHO 2007 body mass index-for-age reference, no participant changed nutritional category; the small number of adolescents, however, limits the precision of this comparison.

GDM classification was retrospective and based on routinely recorded data. Laboratory confirmation was available for 88.2% of cases, indicating that glucose testing was broadly performed; in the remaining six cases, the diagnosis was ascertained solely from the delivery record, which may have led to misclassification. In a sensitivity analysis excluding these cases, the prevalence was 23.8% (95% CI 17.9–30.4%). In addition, because first-trimester fasting glucose was used to identify early GDM, 49.0% of cases were diagnosed at 24 weeks. When only glycemic levels at 24–28 weeks were considered, the prevalence was 10.6% (95% CI 6.5–16.0%), within the range reported in the literature [9,17]. This difference reflects the broader case definition adopted and should be considered when comparing our estimates with those of studies based on standard 24–28-week screening.

This study also has limitations related to the laboratory component. Because samples were shipped to the analysis laboratory in batches after the completion of each collection phase, storage duration at −80 °C prior to biochemical analysis varied among participants, ranging from approximately 1 month to over a year depending on the collection date. Although samples were consistently stored at −80 °C, which is considered stable for the biomarkers analyzed, differential storage duration cannot be fully excluded as a potential source of variability in the results. Additionally, inter- and intra-assay coefficients of variation were not systematically recorded and could not be reported. Finally, biochemical analyses were performed in a randomly selected subsample of 20 participants per group, which, although reduced in size, provided statistical power above 99% or DCF and SOD and approximately 85% for GSH), which constitutes the main focus of this study.

## 5. Conclusions

This study demonstrated the operational feasibility of establishing a prospective mother–child cohort within the real-world setting of primary care units and hospitals in Brazil, in the absence of a dedicated research infrastructure and relying on the collaboration and voluntary engagement of health professionals, while also documenting the magnitude of attrition associated with this design. Recruitment and retention were particularly challenging during the child follow-up phase. Although reasons for non-attendance were not systematically recorded, participants who were reached most frequently mentioned return to work, and the study team perceived caregiver burden and the need to attend appointments with the child as additional barriers. These factors should be formally investigated in future cohort studies conducted in similar settings.

At enrolment, 28.6% of the women had overweight and 24.3% had obesity, meaning that more than half of the cohort (52.9%) was above the recommended weight range. Gestational diabetes mellitus was registered in 51 women (27%). The frequency of pregnancy loss (12.2%) was consistent with previous estimates. These findings underscore the relevance of monitoring nutritional and metabolic status from the pre-conception period and throughout prenatal care. Future analyses from this cohort will examine associations between pre-pregnancy obesity, GDM, oxidative stress biomarkers, and maternal and child health outcomes, which will allow more definitive conclusions about their impact.

## Figures and Tables

**Figure 1:**
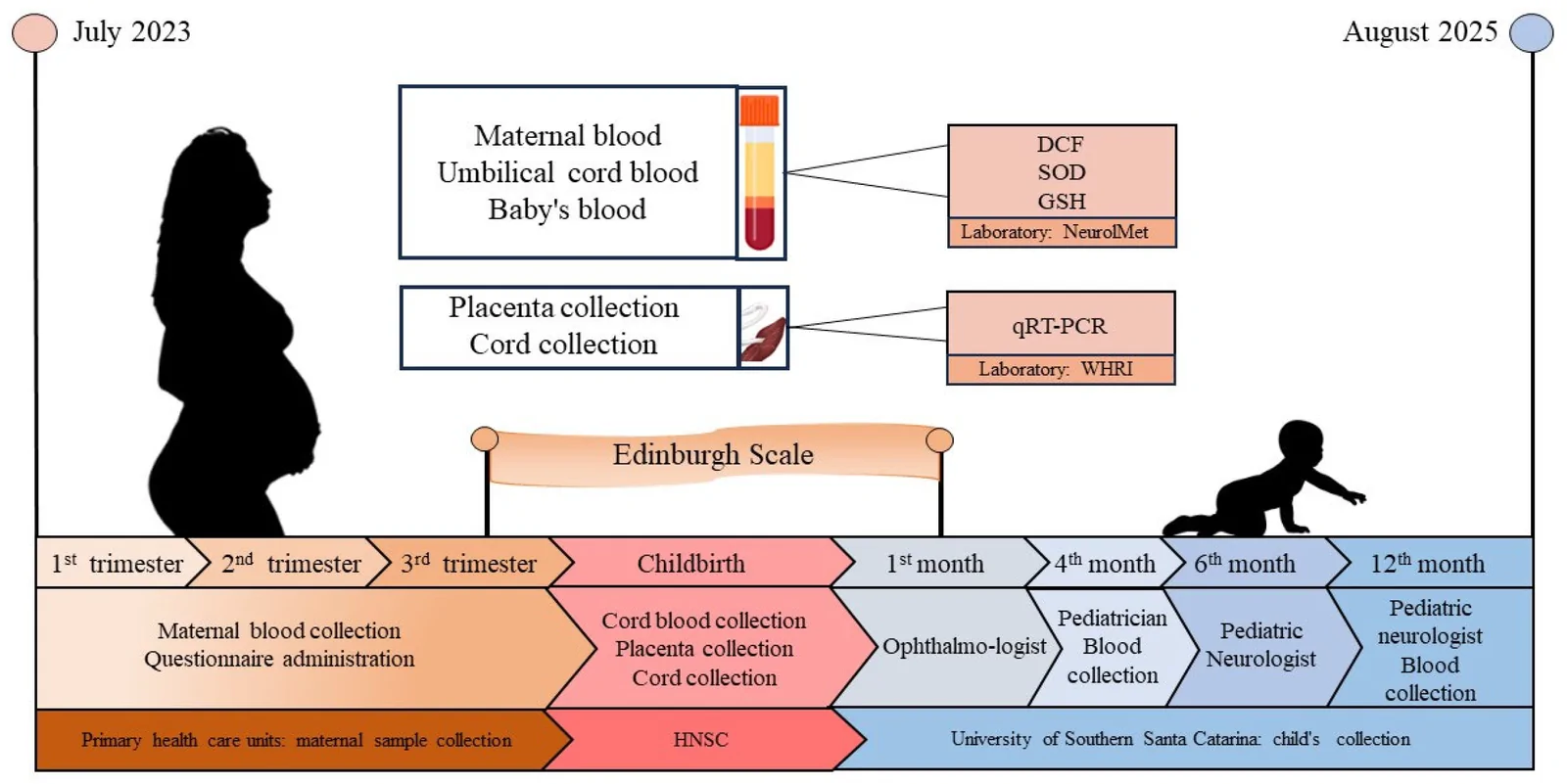
Overview and objectives of the Gerar Cohort, Southern Brazil, 2023–2025.

**Figure 2:**
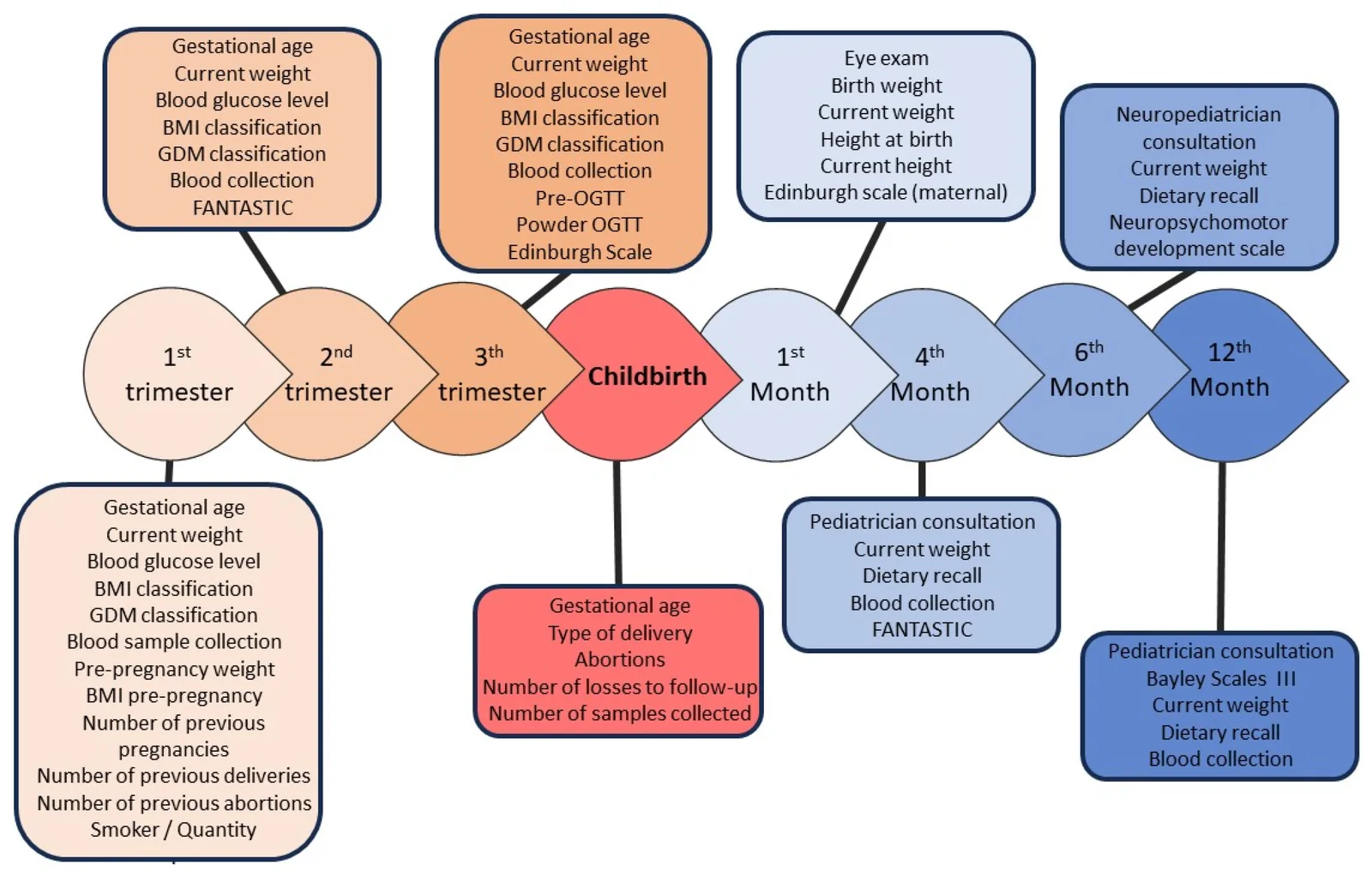
Description of the data collection and evaluation procedures performed at each stage of the study, from the gestational period until the child was 12 months old.

**Figure 3:**
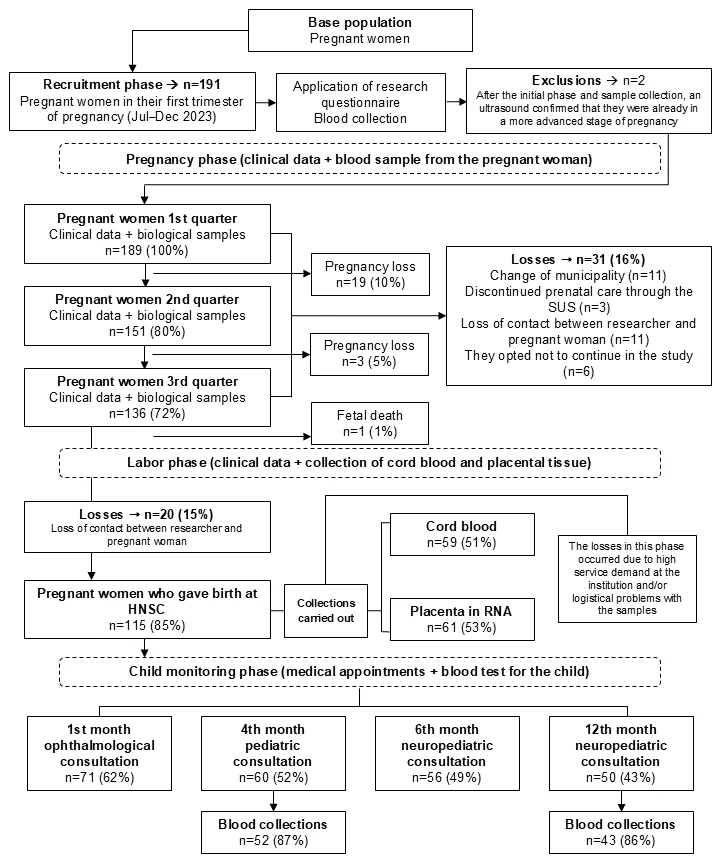
Flowchart of sample behavior by data collection phases. Cohort study, Southern Brazil, 2023–2025 (N = 189). Values are expressed as absolute (*n*) and relative (%) frequencies.

**Table 1:** Sociodemographic and clinical characteristics of pregnant women recruited in the Gerar Cohort, Southern Brazil, 2023–2025 (N = 189). Values are expressed as absolute (*n*) and relative (%) frequencies.

Variable	*n*	% ^1^
Age range		
To 19	20	10.6
20–35	146	77.2
Over 35	23	12.2
Marital status		
Single	123	65.1
Married	64	33.9
Divorced	2	1.1
Number of past pregnancies		
0	64	33.9
1	65	34.4
2	34	18.0
3	15	7.9
4	9	4.8
5	2	1.1
Number of past deliveries		
0	74	39.2
1	68	36.0
2	34	18.0
3	9	4.8
4	4	2.1
Number of abortions in past pregnancies		
0	151	79.9
1	33	17.5
2	4	2.1
3	1	0.5
Smoker		
Yes	14	7.4
No	175	92.6

^1^ Percentages calculated over the 189 participants enrolled.

**Table 2:** Description of the pregnant women participating in the Gerar Cohort, Southern Brazil, 2023–2025, according to the interest groups. N = 189.

Interest Groups	First Trimester	Second Trimester	Third Trimester	Pregnancy Losses ^2^
	*n* (%)	*n* (%)	*n* (%)	*n*
Eutrophy	66 (34.9)	54 (35.8)	46 (33.8)	9
with GDM ^1^	23 (12.2)	20 (13.2)	20 (14.7)	2
Obesity	34 (18.0)	23 (15.2)	20 (14.7)	7
with GDM ^1^	12 (6.3)	12 (7.9)	12 (8.8)	0
Overweight	38 (20.1)	26 (17.2)	22 (16.2)	5
with GDM ^1^	16 (8.5)	16 (10.6)	16 (11.8)	0
Total	189	151	136	23

Note: ^1^ GDM = gestational diabetes. ^2^ The exact gestational timing and cause of pregnancy losses could not be ascertained for all cases; miscarriage and fetal death were therefore analyzed as a combined category.

**Table 3:** Number of biological samples per interest group collected at the time of delivery, at the reference hospital. Gerar Cohort, Southern Brazil, 2023–2025. N = 115.

Interest Groups	Cord Blood	Placenta and Umbilical Cord in RNA ^2^
	*n* (%)	*n* (%)
Eutrophy (*n* = 40)	18 (45.0)	19 (47.5)
with GDM ^1^ (*n* = 15)	11 (73.3)	10 (66.7)
Obesity (*n* = 17)	8 (47.1)	7 (41.2)
with GDM ^1^ (*n* = 10)	4 (40)	5 (50)
Overweight (*n* = 19)	10 (52.6)	11 (57.9)
with GDM ^1^ (*n* = 14)	8 (57.1)	9 (64.3)
Total	59 (51.3)	61 (53.0)

^1^ GDM = gestational diabetes; ^2^ RNA = ribonucleic acid.

**Table 4:** Number of children monitored during the Gerar Cohort, Southern Brazil, 2023–2025. N = 115.

Interest Groups	First Month	Fourth Month		Sixth Month	12th Month	
	Visit ^2^	Visit	Blood Collection	Visit	Visit ^3^	Blood Collection
	*n*	*n*	*n*	*n*	*n*	N
Eutrophy (*n* = 40)	23	17	13	18	17	16
with GDM ^1^ (*n* = 15)	11	14	12	11	5	5
Obesity (*n* = 17)	9	9	9	6	7	7
with GDM ^1^ (*n* = 10)	7	5	5	3	5	3
Overweight (19)	14	9	7	10	8	5
with GDM ^1^ (*n* = 14)	7	6	6	8	8	7
Total	71	60	52	56	50	43

Note: ^1^ GDM = gestational diabetes; ^2^ ophthalmologist; ^3^ neuropediatrician.

**Table 5:** Baseline characteristics of women retained versus lost to follow-up. Gerar Cohort, Southern Brazil, 2023–2025.

	Total Sample	Retained to the Third Trimester	Lost to Follow-Up During Gestational Period	
	N = 189	*n* = 135	*n* = 31	*p*-Value ^1^
Maternal age, median (IQR)	27 (10)	27 (9)	25 (9)	0.167
Age range, *n* (%)				
To 19	20 (10.6)	13 (9.6)	5 (16.1)	0.681
20–35	146 (77.2)	107 (79.3)	23 (74.2)	
Over 35	23 (12.2)	15 (11.1)	3 (9.7)	
Marital status, *n* (%)				
Single	123 (65.1)	89 (65.9)	22 (71.0)	0.283
Married	64 (33.9)	45 (33.3)	8 (25.8)	
Divorced	2 (1.1)	1 (0.7)	1 (3.2)	
BMI, median (IQR)	25.4 (7.22)	25.3 (6.92)	25.4 (7.34)	0.431
BMI classification, *n* (%)				
Eutrophic	89 (47.1)	66 (48.9)	12 (38.7)	0.576
Obesity	46 (24.3)	31 (23.0)	8 (25.2)	
Overweight	54 (28.6)	38 (28.1)	11 (35.5)	
Primigravid women ^2^, *n* (%)	64 (33.4)	53 (39.3)	9 (29.0)	0.288

Note: ^1^ Mann–Whitney U test for continuous variables (non-normal distribution, verified by the Shapiro–Wilk test); chi-square test or Fisher’s exact for categorical variables. Comparisons at the third-trimester stage exclude the 23 pregnancies ending in miscarriage or fetal death (clinical outcomes, not attrition). ^2^ Primigravida was defined as a woman in her first pregnancy. Baseline characteristics were measured at enrollment for all 189 women. Continuous variables are reported as median (interquartile range) due to non-normal distributions. Percentages may not sum to 100 because of rounding.

**Table 6:** Sociodemographic and clinical characteristics of women with delivery data (*n* = 115) and of children followed up to 12 months of age (*n* = 50). Gerar Cohort, Southern Brazil, 2023–2025.

	Retained to the 12-Month Assessment	Not Assessed at 12 Months	
	*n* = 50	*n* = 65	*p*-Value ^1^
Maternal age, median (IQR)	29.5 (8.75)	26 (9.0)	0.005
Age range, *n* (%)			
To 19	9 (18)	2 (3.1)	0.007
20–35	38 (76)	51 (78.5)	
Over 35	3 (6)	12 (18.5)	
Marital status, *n* (%)			
Single	32 (64.0)	45 (69.2)	0.744
Married	18 (36.0)	19 (29.2)	
Divorced	0	1 (1.5)	
BMI, median (IQR)	25.6 (6.86)	24.9 (7.42)	0.848
BMI classification, *n* (%)			
Eutrophic	22 (44.0)	33 (50.8)	0.734
Obesity	12 (24.0	15 (23.1	
Overweight	16 (32.0)	17 (26.2)	
Primigravid women ^2^, *n* (%)	17 (34.0)	29 (44.6)	0.249

Note: ^1^ Mann–Whitney U test or chi-square test, as appropriate; continuous variables are reported as a median (interquartile range) due to non-normal distributions. ^2^ Primigravida was defined as a woman in her first pregnancy. The denominator comprises the 115 live births registered. Percentages may not sum to 100 because of rounding.

## Data Availability

The data presented in this study are available on request from the corresponding author. The data are not publicly available due to privacy and ethical restrictions, as they contain sensitive personal health information from pregnant women and children, in accordance with the study’s ethics approval and Brazilian data protection legislation.

## Supplementary Materials

The following supporting information can be downloaded at https://www.mdpi.com/article/10.3390/healthcare14213783/s1, Table S1: STROBE Statement—Checklist of items that should be included in reports of cohort studies. Title: Obesity and diabetes in pregnancy and maternal and child health: the Gerar Cohort Profile, Brazil. Table S2. Sample size adequacy verification by exposure–outcome combination (OpenEpi^®^, 80% power, α = 0.05). Gerar Cohort Study, Brazil. Notes: *p*_0_ = outcome prevalence among unexposed women (literature estimates). Converted RRs: RR = OR/[(1 − *p*_0_) + (OR × *p*_0_)], with *p*_0_ = 11% (preterm birth) and 17% (LGA); Refs. [19,21] are adjusted ORs. Expected numbers from ~166 eligible women, using obesity 23.4% (≈39) [16] and GDM 8.7–16.7% (14–27) [17]. The achieved sample reflects a census-based strategy; calculations verify adequacy, not recruitment targets.

## Abbreviations

The following abbreviations are used in this manuscript:

ANOVA	Analysis of variance
DCF	Dichlorofluorescein
DNA	Deoxyribonucleic acid
EPDS	Edinburgh Postnatal Depression Scale
GDM	Gestational Diabetes Mellitus
GSH	Reduced glutathione
HDI	Human Development Index
HNSC	Nossa Senhora da Conceição Hospital
IQR	Interquartile Range
LGA	Large-size fetus for gestational age
OGTT	Oral glucose tolerance test
RNA	Ribonucleic acid
ROS	Reactive oxygen species
SOD	Superoxide dismutase
SUS	Government Health System (Sistema Único de Saúde, in Portuguese)
UBS	Primary Health Care Units (Unidades Básicas de Saúde, in Portuguese)
