# Supplementary material for: Obesity and Diabetes in Pregnancy and Maternal and Child Health: The Gerar Cohort Profile, Brazil
Source: Healthcare (Basel). 2026 Nov 5;14(21):3783. doi: 10.3390/healthcare14213783 (PMC13762829; doi:10.3390/healthcare14213783)
Supplement: Supplementary file 1 [file healthcare-14-03783-s001.zip › SUPPLEMENTARY MATERIAL_S2.pdf]

Table S2. Sample size adequacy verification by exposure–outcome combination (OpenEpi®, 80% power, α = 0.05). Gerar Cohort Study.

Exposure	Outcome	Outcome prevalence in unexposed (p_0)	Effect estimate (literature)	Converted RR*	Allocation ratio k (exp:unexp)	Minimum n exposed	Minimum n unexposed	Expected n in cohort (≈166 eligible)	Assessment
Pre-gestational obesity	Preterm birth	11% [18]	OR 2.01 (95% CI 1.44–2.82), adjusted [19]	1.81	1:4	139	553	≈39	Underpowered — exploratory analysis
Pre-gestational obesity	Large-for-gestational-age (LGA)	17%[20]	OR 2.42 (95% CI 2.16–2.72), meta-analysis [21]	1.95[21]	1:4	61	244	≈39	Underpowered — exploratory analysis
Gestational diabetes mellitus (GDM)	LGA	17%[20]	RR 3.29 [22]	3.29 (as reported)	1:6	14	82	14–27	Marginally adequate across the prevalence range; adequate with comfortable margin only at the upper range
Gestational diabetes mellitus (GDM)	LGA	17%[20]	RR 3.29 [22]	3.29 (as reported)	1:8	13	101	14–27	Marginally adequate across the prevalence range; adequate with comfortable margin only at the upper range.

Notes: p_0 = outcome prevalence among unexposed women, from literature estimates. Effect estimates from references [19] and [21] are adjusted odds ratios; *Converted RRs were obtained using: **RR = OR / [(1 − p_0) + (OR × p_0)],** with p_0 = 11% for preterm birth and 17% for LGA. Expected numbers were derived from approximately 166 eligible women (those initiating prenatal care, ~one third of the ~500-women prenatal caseload estimated by the Municipal Health Foundation), applying literature prevalences: obesity 23.4% (≈39 women) [16] and GDM 8.7–16.7% (14–27 women) [17]; co-occurrence of both conditions is possible. The achieved sample resulted from a census-based strategy and was therefore determined by the accessible population; the calculations above serve as adequacy verification for each exposure–outcome combination, not as recruitment targets.
