# Supplementary material for: Obesity and Diabetes in Pregnancy and Maternal and Child Health: The Gerar Cohort Profile, Brazil
Source: Healthcare (Basel). 2026 Nov 5;14(21):3783. doi: 10.3390/healthcare14213783 (PMC13762829; doi:10.3390/healthcare14213783)
Supplement: Supplementary file 1 [file healthcare-14-03783-s001.zip › Supp_S1_STROBE-cohort_Gerar.pdf]

Supplementary Material Table S1

STROBE Statement—Checklist of items that should be included in reports of *cohort studies*

Title: Obesity and diabetes in pregnancy and maternal and child health: the Gerar Cohort Profile, Brazil

	**Item No**	**Recommendation**	**Section/Page**
**Title and abstract**	1	(*a*) Indicate the study's design with a commonly used term in the title or the abstract	1
		(*b*) Provide in the abstract an informative and balanced summary of what was done and what was found	2 (lines 37-58)
**Introduction**			
Background/rationale	2	Explain the scientific background and rationale for the investigation being reported	2/3
Objectives	3	State specific objectives, including any prespecified hypotheses	3 (lines 95-106)
**Methods**			
Study design	4	Present key elements of study design early in the paper	3 (lines 110-112)
Setting	5	Describe the setting, locations, and relevant dates, including periods of recruitment, exposure, follow-up, and data collection	4- 9
Participants	6	(*a*) Give the eligibility criteria, and the sources and methods of selection of participants. Describe methods of follow-up	4-8
		(*b*) For matched studies, give matching criteria and number of exposed and unexposed	NA
Variables	7	Clearly define all outcomes, exposures, predictors, potential confounders, and effect modifiers. Give diagnostic criteria, if applicable	6 + Figure 2
Data sources/ measurement	8*	For each variable of interest, give sources of data and details of methods of assessment (measurement). Describe comparability of assessment methods if there is more than one group	5/6 (lines 216-243)
Bias	9	Describe any efforts to address potential sources of bias	Lines 433-435 + Discussion section (lines 636-640)
Study size	10	Explain how the study size was arrived at	4 (lines 152-161)
Quantitative variables	11	Explain how quantitative variables were handled in the analyses. If applicable, describe which groupings were chosen and why	6/7 (lines 274-293) 10(lines 432-448)
Statistical methods	12	(*a*) Describe all statistical methods, including those used to control for confounding	9/10(lines 433-463)
		(*b*) Describe any methods used to examine subgroups and interactions	6 (lines 248-250; 270-273) 10 (lines 443 – 448)
		(*c*) Explain how missing data were addressed	Figure 3, lines 461-463
		(*d*) If applicable, explain how loss to follow-up was addressed	Figure 3, limitations
		(*e*) Describe any sensitivity analyses	Table 5 and Table 6
**Results**			
Participants	13*	(a) Report numbers of individuals at each stage of study—eg numbers potentially eligible, examined for eligibility, confirmed	Figure 3

		eligible, included in the study, completing follow-up, and analysed	
		(b) Give reasons for non-participation at each stage	Figure 3, text lines 467, 491-499
		(c) Consider use of a flow diagram	Figure 3
Descriptive data	14*	(a) Give characteristics of study participants (eg demographic, clinical, social) and information on exposures and potential confounders	Table 1<br>Text lines 477-479
		(b) Indicate number of participants with missing data for each variable of interest	N/A (cohort profile)
		(c) Summarise follow-up time (eg, average and total amount)	Figure 3<br>Page 16, lines 573-587
Outcome data	15*	Report numbers of outcome events or summary measures over time	Table 2+ 4. Lines 486 - 500
Main results	16	(*a*) Give unadjusted estimates and, if applicable, confounder-adjusted estimates and their precision (eg, 95% confidence interval). Make clear which confounders were adjusted for and why they were included	For main variables: lines 486-490
		(*b*) Report category boundaries when continuous variables were categorized	Lines 477-479, 495, 538
		(*c*) If relevant, consider translating estimates of relative risk into absolute risk for a meaningful time period	N/A (cohort profile)
Other analyses	17	Report other analyses done—eg analyses of subgroups and interactions, and sensitivity analyses	S2
**Discussion**			
Key results	18	Summarise key results with reference to study objectives	Lines 573-596
Limitations	19	Discuss limitations of the study, taking into account sources of potential bias or imprecision. Discuss both direction and magnitude of any potential bias	Lines 661-729
Interpretation	20	Give a cautious overall interpretation of results considering objectives, limitations, multiplicity of analyses, results from similar studies, and other relevant evidence	Lines 731-748
Generalisability	21	Discuss the generalisability (external validity) of the study results	Lines 681-701
**Other information**			
Funding	22	Give the source of funding and the role of the funders for the present study and, if applicable, for the original study on which the present article is based	Lines 757-763

*Give information separately for exposed and unexposed groups.

Note: An Explanation and Elaboration article discusses each checklist item and gives methodological background and published examples of transparent reporting. The STROBE checklist is best used in conjunction with this article (freely available on the Web sites of PLoS Medicine at http://www.plosmedicine.org/, Annals of Internal Medicine at http://www.annals.org/, and Epidemiology at http://www.epidem.com/). Information on the STROBE Initiative is available at http://www.strobe-statement.org.
